# Effect of Low-Dose Aspirin on the Elderly

**DOI:** 10.7759/cureus.54658

**Published:** 2024-02-21

**Authors:** Ali Darraj

**Affiliations:** 1 Department of Medicine, College of Medicine, Shaqra University, Shaqra, SAU

**Keywords:** elderly people, diabetes mellitus, primary prevention, cardiovascular disease, low dose of aspirin

## Abstract

Aspirin is a recognized and affordable antiplatelet medicine. Low amounts of aspirin have been used to prevent cardiovascular events, and it is still widely used for primary and secondary stroke prevention. The purpose of this review article is to evaluate the effects of using low doses of aspirin among elderly people. Although taking large dosages of aspirin (500 mg daily) reduces the long-term risk of colorectal cancer, its effectiveness for long-term prevention may be limited by adverse effects. Studies have assessed the relationship between aspirin dosage, incidence, and death in patients with colorectal cancer. Research has indicated that those with diabetes mellitus have an increased risk of cardiovascular events. Low amounts of aspirin have been used to prevent cardiovascular events. However, there is uncertainty regarding the potential benefits and risks associated with preventing the development of cardiovascular problems in individuals with diabetes. The use of aspirin lowers the risk of occlusive vascular events but raises the possibility of bleeding. More attention should be paid to reducing inappropriate aspirin usage in light of its prevalence, particularly among older persons, as the substantial continuous usage of this drug increases the chances of bleeding.

## Introduction and background

Low amounts of aspirin, as an antiplatelet medication, have been used to prevent cardiovascular events at doses of 75-100 mg/d. Aspirin is one of the earliest and most widely used drugs in the world, and it has long been known that aspirin helps those with a history of cardiovascular disease (CVD) to avoid cardiovascular events [1-3]. However, the overall advantage of aspirin use for CVD primary prevention in individuals with and without diabetes remains controversial. Although people with diabetes have a two to four times higher risk of developing CVD than those without the disease, the effectiveness of aspirin in primary prevention for both groups is less evident. Aspirin is still widely used for primary and secondary stroke prevention despite some recent unfavorable findings. Because CVD is more common in older people, aspirin may have more potential advantages in this age group than it does in younger ones. Nonetheless, older age groups are also known to have an increased risk of bleeding [4,5].

Many countries have experienced a dramatic demographic shift towards an aging society as a result of the significant rise in life expectancy. Therefore, maintaining excellent health in elderly people is a public health goal that is becoming increasingly crucial. As CVDs are one of the leading causes of disability and mortality in the elderly, it is essential that such conditions must be prevented. Although aspirin was originally commercialized in 1899, it is a vital part of antiplatelet therapy for patients with acute coronary syndromes [6-9]. However, the role of low-dose aspirin in primary prevention is still debatable [10], with organizations in Europe [11] as well as North America [12] giving conflicting treatment recommendations. In a more recent statement, the United States Preventive Services Task Force (USPSTF) recommended starting low-dose aspirin use in adults aged 50 to 59 years who have a 10-year CVD risk of ≥10%, are not at increased risk for bleeding, have a life expectancy of at least 10 years, and are prepared to take low-dose aspirin daily for at least 10 years [13]. This guideline is based on growing research showing that low-dose aspirin can prevent colorectal cancer, as well as other types of cancer [9,14].

The therapeutic value of aspirin in lowering cardiovascular ischemic events in atherosclerosis patients is supported by the essential function of platelets in the onset of atherothrombosis [15]. However, studies on specific patients have demonstrated that the antiplatelet efficacy of aspirin varies [16-18]. Patients with insufficient aspirin platelet inhibition are more likely to experience reclusion after peripheral angioplasty or increased myonecrosis after elective percutaneous coronary intervention (PCI) among stable cardiovascular patients, as well as more myocardial infarction, stroke, and cardiovascular deaths [17,19]. It was recommended that the best course of action to enhance the efficiency and reduce the toxic effect of aspirin is to utilize a dose of 50-100 mg on a daily basis [15,20].

Around the world, aspirin is often taken at low doses (referred to as <325 mg/d), especially to avoid CVD [21]. Some societies only advise the use of low-dose aspirin for secondary CVD prevention in people with a 10-year probability of a heart attack or stroke of >10% and who are not at increased chance of bleeding. USPSTF advises that aspirin be taken for primary CVD prevention [22,23]. Although more than 30% of the population of the United States (US) takes aspirin to prevent CVD, recently, its use has most likely declined recently [24]. Aspirin inhibits cyclooxygenase in an irreversible manner, which blocks platelet thromboxane A2 as well as arterial thrombus formation [25]. Low-dose aspirin use has been associated with a lower risk of cancer-related death, as well as other long-term chronic diseases [24].

Although aspirin is currently not recommended for cancer prevention in European or American guidelines [24], the issue remains subject to controversy [26,27]. Aspirin is a recognized and reasonably priced medication for people with CVDs [1]. Despite evidence that the majority of patients benefit significantly from therapy, prescribing in general practice has been regularly acknowledged to be inadequate [28]. Although guidelines have been provided to support healthcare professionals, many individuals who could benefit from aspirin do not receive treatment. Therefore, it is crucial to increase aspirin treatment [29].

Increased vulnerability to hemorrhage, as a clinical trait of older people, may be related to a weakening of the blood vessels [30,31]. The necessity of evaluating preventative measures in adults with a mean age of <70 years is highlighted by the fact that the chance of stroke increases in populations of older people worldwide [32,33]. Meta-analyses and the findings of recent large studies, the majority of which were carried out in populations where the average age is <70 years, provide information about the effectiveness of low-dose aspirin in the primary prevention of stroke [34-36]. Despite some disparities, these results indicate a tendency towards a decline in ischemic stroke, which is being somewhat prevented by an increase in intracerebral and other intracranial hemorrhage. Overall, the incidence of stroke is not significantly affected by low-dose aspirin use [36].

The largest low-dose aspirin randomized controlled study, known with name of Aspirin in Reducing Events in the Elderly (ASPREE) experiment, was designed to examine the relative risks and benefits of this treatment in older age groups [37]. The ASPREE study design included independent adjudication of stroke and hemorrhagic episodes by independent expert panels [38]. Therefore, the study was well suited to assess the relative risks and advantages of low-dose aspirin in a primary prevention setting. 

## Review

The ASPREE experiment began as a primary preventative trial to determine whether taking 100 mg of enteric-coated aspirin regularly would help older persons live longer, healthier lives [39]. Reasonably healthy older individuals from local communities were recruited for the trial, which was carried out in the US and Australia. The main goal was to achieve disability-free life expectancy, which was outlined as living without dementia or permanent disabilities. The occurrence of initial deaths, dementia, and persistent physical impairment led to the establishment of the main composite endpoint. After a median of 4.7 years of follow-up, the use of low-dose aspirin had no discernible effect on the major endpoint compared to the placebo [39].

Effect of aspirin in the elderly and individuals with diabetes mellitus for primary prevention of CVD

In both women and men, CVD is the most common cause of death. The Centers for Disease Control and Prevention (CDC) estimates that approximately 859,000 Americans die from heart disease each year. CVD risk factors include smoking, inactivity, diet, age, overweight, high blood pressure, and diabetes [40]. Average life expectancy has been growing along with a rise in the number of incidents of diabetes in the US, as more people are being diagnosed with the disease and receiving better medical care. Both of these elements have spurred the study of methods to extend disease-free survival, particularly with reference to mortality from CVD. Because of its benefits for the cardiovascular system, one of the more frequently used drugs is aspirin [40]. Low doses of aspirin permanently block the cyclooxygenase-1 (COX-1) enzyme, blocking the synthesis of thromboxane A2 and platelet aggregation [41]. With low-dose aspirin use, the possibility of ischemia in the cardiovascular system, nervous system, and other systems is decreased because it minimizes platelet aggregation and thrombotic blockage. Aspirin's long-lasting inhibition of platelets is most likely what prevents higher doses of the drug from causing higher reductions in thrombotic events [42]. Aspirin is normally taken in modest amounts, and after a few days, the full effect becomes apparent. However, cerebral bleeding (hemorrhagic stroke) and extracranial bleeding (gastrointestinal bleeding) are two of aspirin's most serious side effects [43].

Aspirin in Diabetes Mellitus

Individuals with type 2 diabetes mellitus experience two to three times greater risk of having CVD compared to those without the disease. Sixty-eight percent of people with diabetes over the age of 65 and 16% of those with strokes die from CVD [44]. Such people are more likely to have comorbidities such as diabetes, hypertension, dyslipidemia, obesity, smoking, and a sedentary lifestyle. Either of these situations, when combined with diabetes, increases the patient's risk of developing coronary artery disease or ischemic stroke. Aspirin has been used as primary prevention in a number of tests to assess its hazards and benefits in people with diabetic mellitus [45]. The Prevention of Progression of Arterial Disease and Diabetes trial concluded that there was not enough data to support the use of aspirin as the main way of preventing CVD and mortality in individuals with diabetes. According to the named trial, the primary method of reducing mortality from cardiovascular disease in patients with inadequate consumption of aspirin was not supported by sufficient evidence [45]. Furthermore, a meta-analysis of six studies, including the general population conducted by the Anti-Thrombotic Trialists (ATT), revealed that aspirin lowered the incidence of cardiovascular disease by 12% (95% CI: 6-18) [46].

The effects of aspirin on major cardiovascular incidents are similar in patients with and without diabetes. According to one study, patients who had many CVD risk factors experienced more extracranial bleeding [46]. This result implies that people who are more susceptible to CVD (e.g., those with diabetes) are also more susceptible to the negative effects of aspirin [47].

ASCEND was a randomized experiment that examined the impact of taking 100 mg of aspirin daily as the primary method of preventing CVD. There were 15,480 adults with diabetes with no cardiovascular problems who participated in the trial. The first significant cardiovascular event (i.e., stroke, myocardial infarction, transient ischemic attack (TIA), or fatality from any arterial cause other than intracranial hemorrhage) was the main safety outcome. Patients in the ATT study were not taking any other cardio-protective drugs (e.g., statins or blood pressure medicines) and had a lower risk of CVD than those in the ASCEND experiment [48,49]. As a result of this comparison, ASCEND is consequently more realistic for current management. The investigation also brought up the issues of noncompliance and underestimating the impact of aspirin. Each group's average level of treatment adherence was found to be 70% throughout the duration of the study, which was caused by both a reduction in adherence to the trial aspirin and an increase in the use of non-trial aspirin and other antiplatelet medications [47]. However, analysis of the ASCEND experiment showed that the expected number of major bleeding events brought on by taking aspirin was equal to the anticipated number of vascular events prevented by taking aspirin.

Bleeding is associated with high mortality, and hemorrhagic strokes are usually more fatal and significantly more chronic than ischemic strokes, despite the possibility that cardiovascular and bleeding-related diseases are not comparable [50].

Aspirin in the Elderly

It is believed that aspirin may be more beneficial for people who are older due to their higher risk of CVD. Although aspirin has been widely used as a primary prevention in CVD, there is not much study to support this approach, which is primarily based on preventive trials [37]. In primary prevention trials with large middle-aged or older populations, cardiovascular risk factors were detected or eliminated, and serious vascular events were reduced annually by 0.07%, whereas significant bleeding was increased annually by 0.04% (0.01% and 0.03% for intracranial and extracranial hemorrhage, respectively) [51]. Additionally, it was not obvious at the time whether using aspirin as a major preventative measure would lengthen healthy life expectancy. Another placebo-controlled, randomly allocated ASPREE experience evaluated the mechanism through which aspirin affected the length of healthy elderly people's lives without disabilities. The combined outcome of mortality, dementia, or chronic physical disabilities was the main endpoint that was evaluated in order to appropriately represent the objectives of a healthy elderly person and anticipate the reasons for taking this medicine. The experiment was subsequently terminated early because the rates of the primary outcome were comparable between aspirin and placebo groups. Results from the study demonstrated that low-dose aspirin use among healthy older individuals did not increase disability-free life expectancy, and aspirin users also experienced a greater rate of bleeding [50].

Cardiovascular events and bleeding

ASPREE trial further investigated cardiovascular events and serious bleeding as secondary endpoints. The chosen participants had no obvious CVD, a clinical diagnosis of dementia, illness, and atrial fibrillation, clinically severe physical impairment, high chance of bleeding, anemia, or aspirin restrictions or disability. This secondary endpoint comprised hospitalization for heart failure, fatal or nonlethal stroke, nonfatal myocardial infarction, lethal or nonfatal myocardial infarction, and heart disease. It was considered that aspirin would most likely benefit such CVDs. In the study, aspirin use among older people was associated with a comparable risk of CVD compared to placebo use (10.7% compared to 11.3%, respectively, higher risk with a 95% confidence interval of 0.83-1.08). Additionally, aspirin users had an 8.6% chance of serious hemorrhage, as compared to the 6.8% of the placebo group (higher risk 1.38 with a 95% confidence interval of 1.18-1.62; p<0.001). The cumulative incidence of major bleeding increased gradually over the course of the trial. The aspirin group had a greater risk of cerebral hemorrhage and gastrointestinal bleeding than the placebo group. This result may help to explain why the benefits of aspirin found in this trial were lower than those of previous studies [52]. A meta-analysis of 11 primary prevention trials on low-dose aspirin use for the primary prevention of cardiovascular disorders showed a 22% lower risk of nonfatal myocardial infarction (MI) for those taking aspirin. According to the analysis of this trial, low-dose aspirin use did not significantly reduce all-reason or CVD mortality, but it did reduce nonfatal MI and nonfatal strokes [52].

Aspirin's function in preventing colorectal cancer

The three greatest health catastrophes of the third millennium, colorectal cancer (CRC), coronary heart disease (CHD), and Alzheimer's disease (AD), may all be significantly prevented by aspirin. However, the possibility of other risks, such as GI bleeding, must be considered when using aspirin as a preventative measure for such disorders. For the optimal care of any patient, optimizing the benefit-to-risk ratio of aspirin administration is important, including its potential role in innovative areas such as the prevention of colorectal cancer [53].

Colorectal Cancer

Not surprisingly, healthcare professionals prioritize CRC prevention, as cancer is the fourth most common cause of death, and CRC accounts for approximately 600,000 cases of the annual 7.6 million cancer-related deaths [53]. Taking at least 75 mg of aspirin per day for several years decreased the long-term incidence and death from colorectal cancer. The beneficial effect was greatest for proximal colon malignancies, which are not effectively prevented by screening with colonoscopy or sigmoidoscopy [52]. In a prospective cohort trial involving 74,250 women, aspirin use for >20 years was associated with a 35% reduction in the incidence of CRC (relative risk of 0.65, 95% CI: 0.45-0.94) [54]. Other randomized controlled trials have reported similar results [52,55]. For 7588 participants in two aspirin-related randomized trials, the incidence of CRC was decreased by 26% throughout a >20-year post-trial follow-up period [52,54,56].

Regarding the effect of aspirin on particular disease sites, epidemiological and randomized research has shown consistent results. In a cohort of 27,160 women, aspirin was shown to reduce the risk of proximal colon cancer by 33% (hazard ratio (HR) of 0.65, 95% CI: 0.54-0.78), whereas in a cohort of 301,240 men and women, the risk of distal colon cancer and rectal cancer was decreased by 16% (HR of 0.84, 95% CI: 0.71-0.99) and 24% (HR of 0.76, 95% CI: 0.64-0.90), respectively [57].

According to findings of randomized trials by Rothwell et al. [52], aspirin had a greater effect on the proximal colon compared to other areas, with a 55% reduction in CRC incidence (HR of 0.45, 95% CI: 0.28-0.74). This reduced incidence was greater than that observed in either the rectum or distal colon (HR of 0.90, 95% CI: 0.63-1.30). Increased aspirin use has been linked to improved survival after a CRC diagnosis in both epidemiological and randomized studies. According to an early cohort analysis of 662,424 men and women, those who took aspirin more than 16 times per week experienced 30% and 42% reductions in the risk of death from colon cancer [58]. Regular aspirin users showed reductions in CRC mortality of 29% (HR of 0.71, 95% CI) and overall mortality of 21% (HR of 0.79, 95% CI).

A recent cohort analysis of 1279 individuals included men and women with colorectal cancer diagnoses. The results of a cohort study show that taking aspirin on a regular basis after receiving a colorectal cancer diagnosis is linked to a decreased risk of both overall and colorectal cancer-specific death, particularly in those with tumors that overexpress cyclooxygenase-2 [59]. According to an analysis of four randomized trials (n=14,033), over a 20-year follow-up period, CRC mortality was reduced by 34% (relative risk (RR) of 0.66) [52]. In the Cancer and Leukemia Group B (CALGB 89803) trial study, aspirin use was found to reduce CRC mortality by 48%, in which 830 patients with stage III CRC were randomized to receive adjuvant therapy [26]. The advantages of aspirin use in CRC can also apply to many other cancer types. According to an analysis of eight trials (n=25,570), the 20-year risk of any cancer death was reduced by 20% (HR of 0.80, 95% CI) and GI cancer deaths by 35% (HR of 0.65, 95% CI) [60]. Increased duration of aspirin daily use was also linked to further benefits. The HR for CRC mortality was 0.54 for scheduled aspirin use lasting >2.5 years and 0.48 for >5 years, and the HR for any cancer mortality was 0.79 for 5-7.4 years and 0.69 for >7.5 years [52,60]. A number of randomized trials have shown that aspirin can help reduce the risk of recurring colorectal adenoma at an earlier disease stage. According to a pooled analysis of these trials (n=2967), aspirin use over a median 33-month period reduced the risk of any lesion by 17% (RR of 0.83, 95% CI) and advanced lesions by 28% (RR of 0.72, 95% CI) [61-63].

Furthermore, very high-risk patients with a hereditary propensity for numerous adenomas and carcinomas (e.g., carriers of Lynch syndrome) may especially benefit from aspirin. Aspirin was shown to reduce the risk of Lynch syndrome cancer by 38% in a study of 667 carriers (HR of 0.62, 95% CI). The strength of the effect depended on the period of treatment, which is consistent with studies of patients with sporadic CRC. According to outcomes, the effects in individuals who took aspirin (or aspirin placebo) for at least two years, which was defined as consuming 1400 (300 mg) tablets (approximately down from a two-year total of 1461 pills) to accommodate for the occasional missed dosage or early scheduling of the exit colonoscopy. According to this criterion, 258 people (or 60% of those taking aspirin) and 250 people (or 58% of those taking a placebo) received treatment for at least two years. For individuals using aspirin for two years or more, the hazard ratio was 0.41 (95% CI: 0.19-0.86) [64].

A comprehensive assessment of dosing for chemoprevention was conducted [65]. According to the available research, aspirin can inhibit thromboxane A2, a protein involved in the proliferation of tumor cells, at low doses (75 mg/day), which are widely used to prevent CVD. Therefore, anti-neoplastic effects may be evident at relatively low doses. Low doses of aspirin (75-300 mg/day) were linked to a 40% reduction (odds ratio (OR) of 0.60) in the long-term risk of CRC-related mortality in one meta-analysis of randomized trials [52]. This was equivalent to the 28% reduction (OR of 0.72) linked with high aspirin doses (500-1200 mg/day). According to an epidemiological study of 47,363 men with a follow-up of 18 years, the relative risk of CRC was lower in those who used fewer aspirin tablets per week (0.5-1.5 standard aspirins/week) compared with those who used >14 aspirin tablets per week (RR of 0.94 vs. 0.30, respectively). These findings suggest that dose interval may have a more significant impact than dose strength, and it is also concluded that utilizing every-other-day dosing regimens supports this possibility. Overall, these findings show that low-dose aspirin use over multiple years is linked to a reduction in CRC incidence and mortality [53]. The selected studies and main conclusions are shown in Table 1.

**Table 1:** Synthesis of data from the included studies in this review

No	Authors name	Study type	Objective	Conclusion
1	Calderone et al. 2022 [34]	Systematic review and meta-analysis	This study focused on age as a treatment variable to examine the effectiveness and safety of aspirin in people without overt cardiovascular disease.	Aspirin use was linked to a somewhat lower risk of major cardiovascular events and a neutral effect on all-cause mortality in people without overt cardiovascular disease but at the cost of a higher risk of major bleeding. Younger people may benefit from aspirin more significantly.
2	Judge et al. 2020 [35]	Meta-analysis	To look into the effect of aspirin for primary avoidance of cardiovascular events.	In 11 trials (157,054 individuals), aspirin has been linked to a higher chance of hemorrhagic stroke but not a statistically important reduction in nonfatal stroke. There was no statistically significant reduction in mortality risk linked with aspirin.
3	Patel and Baliga, 2020 [50]	Review	To examine the clinical data on the effectiveness of aspirin for individuals with diabetes mellitus and elderly people with good health.	The risks associated with bleeding outweighed the advantages of aspirin treatment in adults with diabetes who had never had cardiovascular disease. In addition, aspirin use in healthy elderly people increased the risk of severe hemorrhage rather than extending disability-free life expectancy.
3.	Bowman et al. 2019 [48]	Randomized controlled trial	To evaluate the effect of aspirin in patients suffering from diabetes mellitus.	Cardiovascular events are linked to an elevated risk in those with diabetes mellitus. The balance of advantages and risks for the prevention of first cardiovascular events in patients with diabetes is uncertain. The use of aspirin lowers the risk of occlusive vascular events but raises the risk of bleeding.
4.	McNeil et al. 2018 [39]	Randomized controlled trial	To analyze the scientific approach to ascertain the effect of aspirin on mortality from all causes in healthy elderly.	When elderly people were taking daily aspirin compared to those taking a placebo, they experienced higher all-cause mortality rates, which were mostly due to cancer-related deaths.
5	Guirguis-Blake et al. 2016 [36]	Review	The goal of this study was to evaluate a systematic study on aspirin's advantages in cardio-vascular event prevention at individual's age of 40 years or more.	Aspirin has a moderately positive effect on the primary prevention of cardiovascular disease when taken daily in doses of 100 mg or less. Older persons appear to gain benefits more relatively from myocardial infarction.
6	García Rodríguez et al. 2016 [43]	Review	To investigate the potential risk of bleeding for a long time from the use of low-dose aspirin.	Low-dose aspirin has similar risks for severe bleeding in real-world situations as it does in randomized studies. The use of low-dose aspirin in the prevention of cardiovascular events will be guided by these facts in clinical decisions.
7	ASPREE Investigator Group 2013 [38]	Randomized controlled trial	To determine if the potential risks of low-dose aspirin use in older, healthy people outweigh any possible primary preventive benefits.	Aspirin's potential to prolong healthy, independent aging in older individuals in the US and Australia is better captured by ASPREE's distinctive composite primary endpoint.
9	Rothwell et al. 2010 [52]	Randomized trials	To examine the long-term effect of aspirin in patients with colorectal cancer.	At least 75 mg of aspirin should be taken every day for several years to prevent the long-term incidence and mortality from colorectal cancer. The highest benefit was seen for proximal colon malignancies, which are not otherwise effectively prevented.

Discussion

Aspirin as a Primary Preventative Measure

Aspirin, at low doses, was still more commonly recommended for primary protection against CVD in 2018 compared to secondary preventive measures. In one study, 3.6% of the adult population reported taking aspirin despite a confirmed diagnosis of atherosclerotic cardiovascular disease (ASCVD). Meanwhile, aspirin was used as a primary preventative measure by more than 20% of adults who were 80 years or older; this group also had a significant annual risk of bleeding (detected in the hospital) of 2.2% [66].

Several other recent studies have investigated aspirin use among the general population. Among a recent cohort of respondents to the self-reported US 2017 National Health Interview Survey (NHIS) in 2017 (n=14,328), consumption of aspirin fluctuated between 7% among those in their 40s and 50s to 46% among those in their 80s or older [67]. The NHIS characterized the primary preventive consumption of aspirin as having no self-identified increase in coronary heart disease, MI, angina, or stroke. The prevalence rates of aspirin use in the US in 2017 were significantly higher than non-aspirin users, where <2% of those aged 40-50 years and 20% of those in their 80s used aspirin that year. This discrepancy may be somewhat attributed to over-the-counter unregistered usage in Denmark, particularly during the start time in the research cycles, but differences in prescription behavior are far more likely to be responsible. As a result, perspectives on the risk-benefit ratio of aspirin varied in the 2010s across Europe and the US, as previously indicated. In Denmark, the proportion of 80-year-olds who regularly took aspirin for preventive purposes was 36% in 2010 and was reduced to almost half of that by 2018. The lower percentage of aspirin usage in our findings relative to NHIS data could be due to variations in registration statistics, self-reported diagnoses, and aspirin consumption, particularly because the NHIS had an aggregate response rate of only 53% in 2017 [67]. Registry-based research that involves the entire population, which relies more on pharmaceutical data as well as physician-diagnosed ASCVD based on international statistical classification (ICD) of diseases and related health problems, is presumably more reliable and moderate, leading to more modest estimates of prevalence.

According to the meta-analysis that included people with and without diabetes, aspirin had a risk reduction ratio of 11% (95% CI: 6-16%) when used for the initial prevention of acute CVDs, in addition to an increase in the relative risk of serious incidents of bleeding of 43% (95% CI = 30%-56%) [5]. In light of the most recent research, the widespread use of aspirin for primary prevention of CVD suggests that a significant portion of it may be ineffective and should be eliminated, ideally based on a mutual preference among patient and physician [68]. Nevertheless, it might be difficult to decide whether one should begin, continue, or stop using aspirin for main preventive purposes in and by particular individuals. Our findings demonstrate that aging generally increases the risk of bleeding. As a result, the most recent US regulations prohibit patients over 70 from taking aspirin on a regular basis but provide little guidance on whether any older patients, without stated ASCVD, need to be prescribed aspirin [67].

Those with type 2 diabetes mellitus make up another significant and difficult group of patients. The existing evidence from clinical trials in these patients has historically been interpreted differently according to European and American criteria [69]. With a 7.4-year average follow-up, the recent ASCEND trial [70] demonstrated that among those with type 2 diabetes, a 0.9% higher rate of severe bleeding events (4.1%) with aspirin vs. with placebo (3.2%) was balanced by a 1.1% absolute reduction in significant vascular events (8.5% and 9.6%, respectively). Additionally, those with a lower risk of vascular events were found to have the optimum risk-benefit ratio. In comparison, the cohort with the greatest chance of ASCVD (control five-year risk of major vascular incidents 10%) had a lower risk of significant vascular events. The reduction in severe incidents of bleeding was significantly greater than the prevented number of fatalities from CVDs [70]. According to the latest European and American criteria on the consumption of antiplatelet medications for patients with type 2 diabetes, low-dose aspirin may be used as a main preventative measure in patients at significant/very high cardiovascular threat, in the lack of obvious contraindications or in those who have a higher cardiovascular risk after discussing the advantages and disadvantages (greater possibility of bleeding) with the patient [71,72]. To assist doctors and patients in evaluating the actual effect of aspirin use and the advantages of aspirin for protection purposes based on unique patient features, there seems to be an enormous desire for clear, encouraging guidelines and easily accessible data.

## Conclusions

Aspirin is a recognized and affordable antiplatelet medicine. Elderly people are more likely to utilize low-dose aspirin as a main preventative measure to avoid atherosclerotic circulatory conditions. More attention should be paid to reducing inappropriate aspirin usage, as it is currently widely used, particularly among older persons, and the substantial continuous use of this drug increases the risk of bleeding. Studies showed that the risks associated with bleeding outweighed the advantages of aspirin treatment in adults with diabetes who had never had CVD. In addition, aspirin use in healthy elderly people increased the risk of severe hemorrhage rather than extending disability-free life expectancy.

Aspirin is the primary antiplatelet medicine for the treatment of CVD, particularly in patients at high risk, and is now advised to be used in low doses, usually once daily. Loss of blood is not more likely to occur in individuals having an elevated risk of CVD for more than a decade. Such people should be prepared to take aspirin at a low dose every day for at least 10 years and have a minimum life expectancy of 10 years. This recommendation is supported by expanding evidence that low-dose aspirin can help prevent colorectal cancer and other cancers.
